# MicroRNA-21-containing microvesicles from tubular epithelial cells promote cardiomyocyte hypertrophy

**DOI:** 10.1080/0886022X.2021.1891098

**Published:** 2021-02-26

**Authors:** Jia Di, Min Yang, Hua Zhou, Min Li, Jiabi Zhao

**Affiliations:** a Department of Nephrology, The First People's Hospital of Changzhou, Changzhou, China; b Department of Pathology, The Second People's Hospital of Changzhou, Changzhou, China

**Keywords:** MicroRNA, microvesicle, cardiomyocyte hypertrophy, chronic kidney disease, cell-cell communication

## Abstract

**Background:**

Cardiomyocyte hypertrophy has been reported as one of the important mechanisms for cardiovascular disease (CVD) in patients with chronic kidney disease (CKD). MiroRNA-21(miR-21) was determined to play an important role in myocardial hypertrophy. However, the role of microvesicles (MVs) containing miR-21 in CKD-related cardiomyocyte hypertrophy remains largely unexplored.

**Methods:**

Renal tubular epithelial cells were stimulated by transforming growth factor (TGF-β1), and the conditioned medium was extracted by differential centrifugation. Renal tubular epithelial cells were labeled with Dil-C18 dye and the recipient cardiomyocytes were observed by fluorescence microscope. MiR-21 level in MVs was detected by qRT-PCR, and the length and diameter of cardiomyocytes were measured by microscope. BCA protein kit and ANP kit were used to detect the content of cell protein and the level of ANP. MiR-21 inhibitor was transfected into cardiomyocytes to observe the effect of miR-21 on myocardial hypertrophy.

**Results:**

TGF-β1 could induce donor renal tubular epithelial cells to produce MVs and delivered into cardiomyocytes, followed by the diameter, protein concentration and ANP content of cardiomyocytes significantly increased. Meanwhile, MiR-21 levels were markedly increased in MVs isolated from donor renal tubular epithelial cells and recipient cardiomyocytes. Pre-transfection of miR-21 inhibitors could inhibit MV-induced cardiomyocyte hypertrophy.

**Conclusion:**

Tubular cells could secrete miR-21 by MVs and deliver it into recipient cardiomyocytes to induce cardiomyocyte hypertrophy. It might shed a new light on the mechanism and treatment of CKD-related cardiac dysfunction.

## Introduction

Cardiovascular events are the leading cause of death among CKD patients [1]. Myocardial remodeling is an important pathological mechanism in the development of heart failure with cardiomyocyte hypertrophy as the main feature. Suppression of cardiomyocyte hypertrophy has been regarded as an effective therapeutic regimen to prevent heart failure in CKD patients. The inhibitors of renin-angiotensin-aldosterone system (RAAS) system can reverse the left ventricular hypertrophy and improve the function of the left ventricle, while such inhibition of RAAS system may also lead to a decrease of glomerular filtration rate and an increased risk of hyperkalemia. Other therapeutic regimens, such as regulating blood flow dynamics, relieving fluid overload, correcting anemia or malnutrition, and correcting bone mineral metabolism disorder, are still lack of the capability for completely reversing heart failure in the CKD. To the best of our knowledge, there are no effective therapies specific for types 3 and 4 Cardiorenal syndrome(CRS) so far [2], which makes it essential to seek potential therapy by exploring other factors involved in CKD-related CVD.

MicroRNAs (miRNAs) play an important role in hypertrophy and apoptosis of cardiomyocytes, and have been used as new biomarkers and therapeutic targets for diagnosis, treatment and prognosis of heart failure [3,4]. In recent years, there are rapidly growing attentions paid on a way of intercellular communication, namely ‘microvesicle (MV)’ [5,6]. Cells can produce little MVs under the normal physiological conditions, while the production of MVs is significantly increased under the pathophysiological conditions. In 2007, Valadi has found that MV contains miRNA [7]. It has been found that a large number of the most basic processes of cell metabolism, proliferation, differentiation and death in physiological and pathological states are regulated by miRNAs [8]. The role of MV containing miRNA for intercellular communication has become a new research hotspot. Moreover, MiRNA-21 (miR-21) has been shown to play an important regulatory role in the progression of chronic cardiac and renal diseases. It is interesting to note that MVs containing miR-21 are significantly increased during myocardial hypertrophy, and most of MVs are contained in the fluid around the heart [9]. However, the origin of these MVs still remains largely unknown. In the present study, we aimed to investigate the role of miR-21-containing MVs from tubular epithelial cells in promoting cardiomyocyte hypertrophy with CKD.

## Materials and methods

### Cell culture and treatment

Rat renal proximal tubular epithelial cells (NRK-52E) and rat cardiomyocyte cells (H9C2) were purchased from ATCC (Manassas, VA). Cells were maintained in Dulbecco’s modified Eagle’s medium (DMEM/F12), cultured with 5% CO2 and 95% humidity, supplemented with 10% fetal bovine serum. After 24 h, NRK-52E cells were cultured in FBS-free medium for another 16 h, then incubated with or without recombined human transforming growth factor β1 (rhTGF-β1) (R&D Systems, MN) at a dose of 5 ng/mL [10]. Donor NRK-52E cells were incubated with rhTGF-β1 for 48 h and maintained in FBS-free DMEM/F12 for another 48 h in experimental group. To generate conditioned medium, rhTGF-β1-free medium was collected and sequentially centrifuged at 300, 1,200 and 10,000 g for 5, 20 and 30 min, respectively. The control medium was generated in the same way but without exposing to rhTGF-β1 in the first 48 h. Then the recipient cardiomyocytes were incubated with control or conditioned medium and collected for further characterization.

### Western blotting analysis

The preparation of whole cell lysates and western blot analysis of protein expression were performed according to the previous routine procedure [11]. In brief, total protein was extracted from H9C2 cells and protein concentration was determined using the Bicinchoninic Acid protein detection kit (Sigma-Aldrich). The whole cell lysis buffer was mixed with SDS loading buffer. After centrifugation at 13,000 g at 4 °C for 20 min, the supernatants were collected. Equal amounts of proteins (50 μg) were subjected to SDS-PAGE on 10 or 5% pre-casted gels (Bio-Rad, Hercules, CA, USA). The supernatant was collected after centrifugation at 13,000 g at 4 °C for 20 min. According to the previously established protocol, protein expression was detected [11]. The primary antibodies used were as follows: anti-SORBS2 (1:200, SAB4200183, Sigma-Aldrich), anti-ENH (1:200, SAB2101761, Sigma-Aldrich) and anti-actin (1:500, A5385, Sigma-Aldrich). The secondary antibody used were anti-mouse IgG (1:10000, A5385, Sigma-Aldrich), anti-rabbit IgG (1:10000, B7389, Sigma-Aldrich). Quantification was performed by measurement of the intensity of the signals with aid of ImageJ (NIH, Bethesda, MD, USA).

### MV isolation

According to a previously described method [7], MVs were separated from the conditioned medium by differential centrifugation. Briefly, cells were centrifuged at 300, 1200, 10,000, and 110,000 g for 5, 20, 30 min and 1 h (all at 4 °C). Then, MVs were collected and resuspended, then isolated total RNA of MVs for further characterization.

### Transmission electron microscopy (TEM)

First, using a droplet of 2.5% glutaraldehyde in PBS, MV pellet was placed and fixed. Then the sample was fixed at 1% osmium tetroxide and embedded, fixed, segmented into several blocks (<1 mm^3^). After being dehydrated by an increased concentration of alcohol, the sample was soaked in an increased concentration of QuetoL-812 epoxy resin mixed with propylene oxide. Then in a pure, fresh quetol-812 epoxy resin, the sample was embedded and polymerized. Using a Leica (Solms, Germany) UC6 ultramicrotome, the sample was cutted into sections (100-nm thick) and post-stained with uranyl acetate and lead citrate for 10 min and 5 min at room temperature respectively. Using an FEI Tecnai T20 transmission electron microscope the sample was observated, operated at 120 kV.

### Fluorescent labeling of MVs and fluorescence microscopy

NRK-52E cells were labeled with Dil-C18 (a lipid dye used to label cell membranes) for 1 h and washed with PBS for 3 times. The medium was collected and the MVs was obtained by centrifugation. MVs were resuspended in DMEM/F12 and incubated with cultured recipient cardiomyocytes. After different incubation times, five non-overlapping fields were observed under a digital camera-equipped Nikon Eclipse 80i Epi-fluorescence microscope. The immunofluorescence images were captured under the same exposure Settings.

### Quantitative real-time PCR (qRT-PCR)

According to the manufacturer’s instructions (Invitrogen), total RNA was prepared using TRIZol reagent and first strand cDNA was synthesized by RT II kit (Qiagen, Düsseldorf, Germany). Subsequently, using an Applied Biosystems 7300 Sequence Detection system, qRT-PCR was performed. The reaction system was follows: 10 µL RNase-free water (Qiagen),1 µL cDNA, 2 µL 10 × miScript Primer Assay, 2 µL 10 × miScript Universal Primer and 10 µL 2 × QuantiTect SYBR Green PCR Master Mix. PCR conditions were as follows: 95 °C for 10 min, 40 cycles at a melting temperature of 95 °C for 15 s, followed by 60 °C for 1 min. All primers were synthesized by Qiagen, and housekeeping gene was U6. All experiments were performed in triplicate. The C_T_ data were determined using default threshold settings, and the mean CT was calculated from the triplicate PCRs. ΔCT = CT treatment－CT control.

### RNA transfection

According to the manufacturer’s instructions, we used Liptofectamine 2000 reagent (Invitrogen) to transfect miRNA-21 mimics or inhibitors and their negative control (NC) RNA (purchased from Qiagen) into H9C2 cells. After transfection, cells were maintained at 37 °C with 5% CO2 and 95% humidity for 24 h, then they were incubated in different conditions until they were ready for further treatment or assay. 

### Measurement of atrial natriuretic peptide (ANP) concentration

H9C2 cells were cultured under different conditions, then removed the culture medium and washed twice with PBS, digested by trypsin. The cells were centrifuged at 800 g, and resuspended with 200 µL PBS and transferred to a new centrifuge tube. The centrifuge tube was placed in an ice bath and lysised. The ANP concentration was determined according to a Rat ANP ELISA Kit (CUSABIO). All experiments were performed in triplicate.

### Measurement of the diameter of cardiomyocyte

H9C2 were cultured under different conditions, digested by trypsin and resuspended. 2 × 10^5^ cells were seeded into the 6-well plate, respectively. After 24 h of cell culture, the medium was replaced with DMEM medium containing 2% fetal bovine serum for further culture for 24 h. The diameters of cardiomyocytes were measured by eyepiece microscale. 6 cardiomyocytes with clear morphology were randomly selected from each group to measure the cell diameter by two experimenters independently. All experiments were performed in triplicate.

### Statistical analysis

Western blotting analysis, RT-qPCR and immunofluorescence staining were all independently repeated at least three times. Continuous variables with normal distribution were compared using an unpaired t-test and the results were presented as mean ± standard deviation (SD). Comparison between groups was conducted using one-way ANOVA (using Dunnett’s test to compare all groups with a single control; using Tukey’s of Bonferroni test to compare comparisons vs. control and MV). All statistical analyses were conducted in SPSS 25.0 software. A two-sided *p* value <0.05 was considered to be statistical significance.

## Results

### MVs derived from TGF-β1-treated tubular cells mediate recipient cardiomyocyte hypertrophy

**Figure 1.**
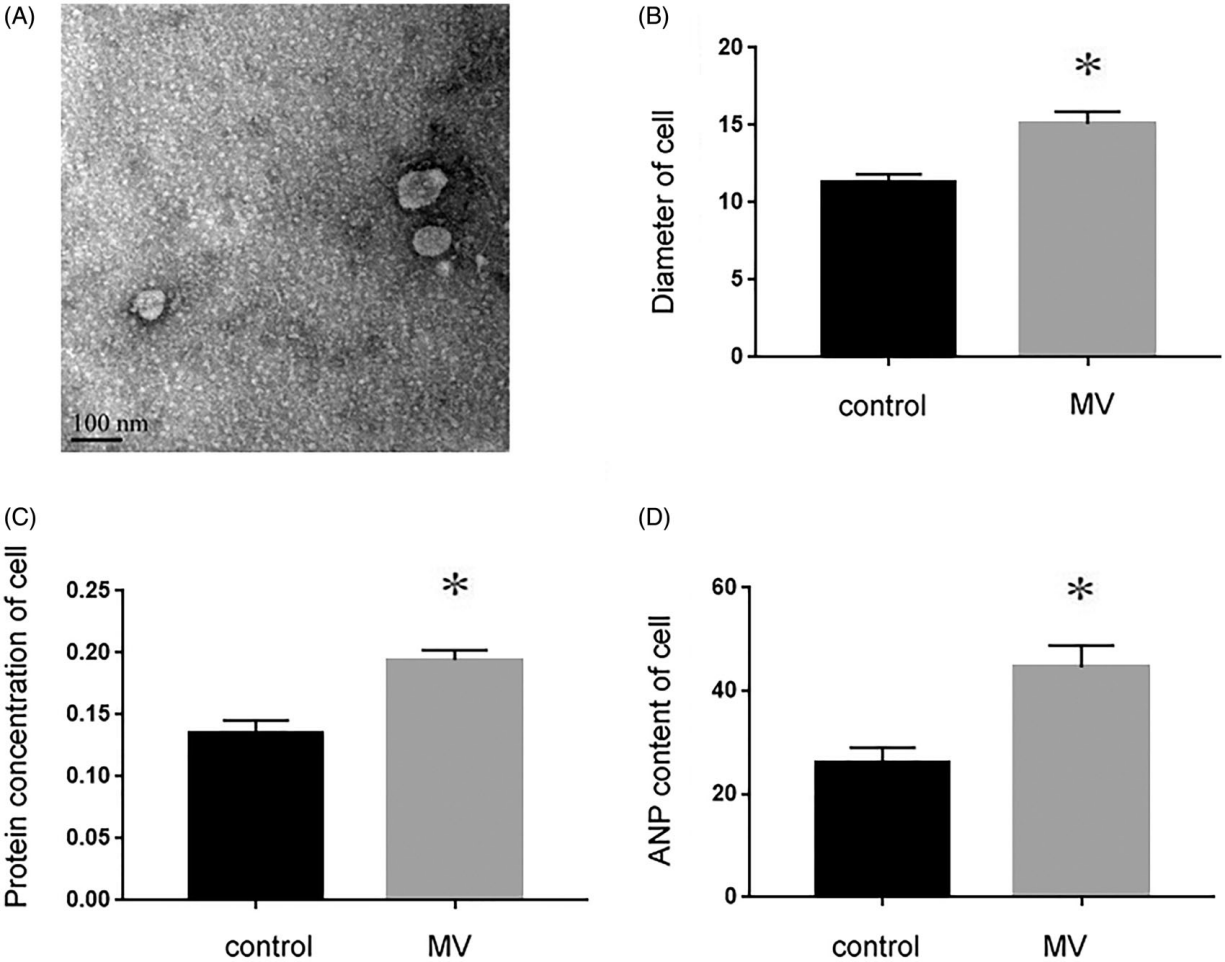
NRK-52E cells incubated with TGF-β1 mediate recipient cardiomyocyte hypertrophy. (A) Electron microscopy image of NRK-52E-derived MVs, showing a size of approximately 60 to 80 nm in diameter. ×10k, Scale bar: 100 nm (*n* = 5). (B) The size of cardiomyocyte was measured with or without the incubation of MVs. (C) Protein concentration was measured with or without the incubation of MVs. (D) ANP content was detected with or without the incubation of MVs. *p < 0.05 versus control.

Recent studies have shown that miRNAs secreted by MVs can serve as paracrine intercellular signaling molecules in different cell types, highlighting their potential role in cell-to-cell communication between kidney and heart. Therefore, we first examined whether injured tubular cells were capable of promoting MVs. NRK-52E is a kidney proximal tubular epithelial cell line of normal rat origin. Figure 1(A) shows that after NRK-52E cells were incubated with TGF-β1 for 48 h, the production and secretion of MVs were assessed by TEM. Our results indicated that compared with control, recipient cardiomyocytes were induced to undergo hypertrophy, evidenced by enlarged cell size (*p* < 0.05, Figure 1(B)), increased protein concentration (*p* < 0.05, Figure 1(C)) and elevated atrial natriuretic peptide (ANP) content (*p* < 0.05, Figure 1(D)).

### MVs labeled with Dil-C18 are transported to cardiomyocytes

**Figure 2.**
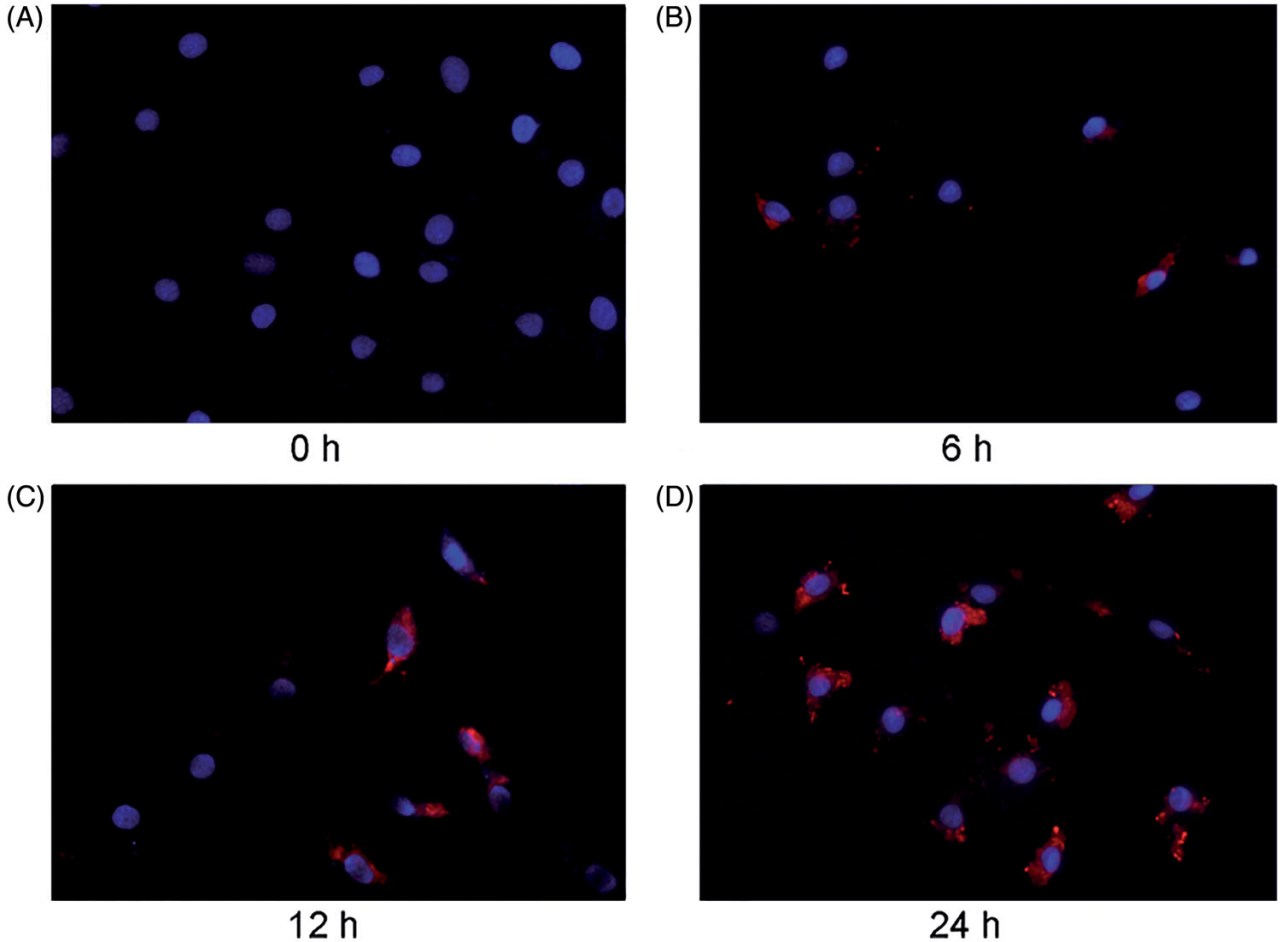
NRK-52E-derived MVs are labeled with orange fluorescent dye and incubated with cardiomyocytes. Microscopy image shows the internalization of fluorescently labeled MVs into cardiomyocytes. Donor NRK-52E cells were labeled with Dil-C18 (red). Control (A), cardiomyocytes incubated with MVs for 6 h (B), 12 h (C) and 24 h (D). ×400.

We next examined whether MVs could enter the recipient cells. NRK-52E cells were labeled with Dil-C18, and the conditioned medium after TGFβ-1 treatment was collected to obtain MVs by ultracentrifugation. Subsequently, MVs were applied to treat recipient cardiomyocytes for indicated durations. Figure 2(A–D) exhibits that Dil-C18-labeled MVs entered the recipient cardiomyocytes in a time-dependent manner. Red fluorescent positive labeled MVs were observed in the recipient cardiac myocytes, and the number increased with the extension of treatment time, suggesting that MVs could be transferred from TGF- β1 treated renal tubular epithelial cells to the recipient cardiac myocytes.

### Up-regulated miR-21 from TGF-β1-treated tubular cells is packaged into MVs and delivered into cardiomyocytes

**Figure 3.**
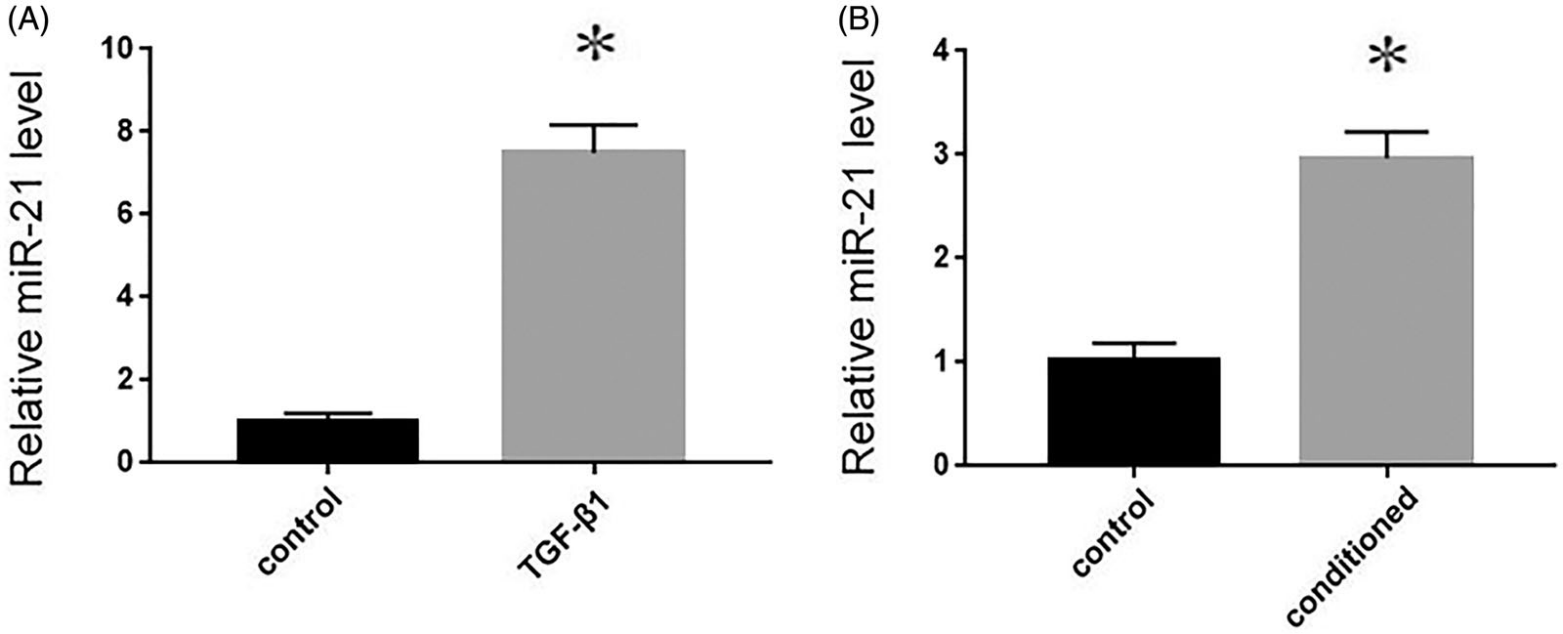
Exogenous miR-21 is delivered by MVs into recipient cardiomyocytes. RT-qPCR of relative miR-21 expression in NRK-52E cells in the absence or presence of 5 ng/mL of TGF-β1 for 12 h. Results are presented as means ± SE of three independent experiments. (A) RT-qPCR of relative miR-21 expression in recipient cardiomyocytes. Results are presented as means ± SE of three independent experiments. (B) *p < 0.05 versus control.

It has identified that miR-21 is temporally up-regulated in obstructive kidneys and TGF-β1 stimulated tubular cells. MiR-21 could be secreted by tubular cells and delivered into recipient tubules by MVs [8]. In the present study, MVs were collected from control or conditioned medium, and then we extracted total RNA from the isolated MVs. Compared with the control medium, the miR-21 expression was markedly increased in MVs isolated from conditioned medium (*p* < 0.05, Figure 3(A)). Thereafter, whether exogenous miR-21 could be packaged by MVs and delivered into recipient cardiomyocytes were further determined. MVs isolated from conditioned medium of donor NRK-52E cells were incubated with recipient cardiomyocytes for 24 h, Similarly, the miR-21 expression was markedly increased in recipient cardiomyocytes (*p* < 0.05, Figure 3(B)).

### Exogenous miR-21 delivered by MVs promotes recipient cardiomyocyte hypertrophy

**Figure 4.**
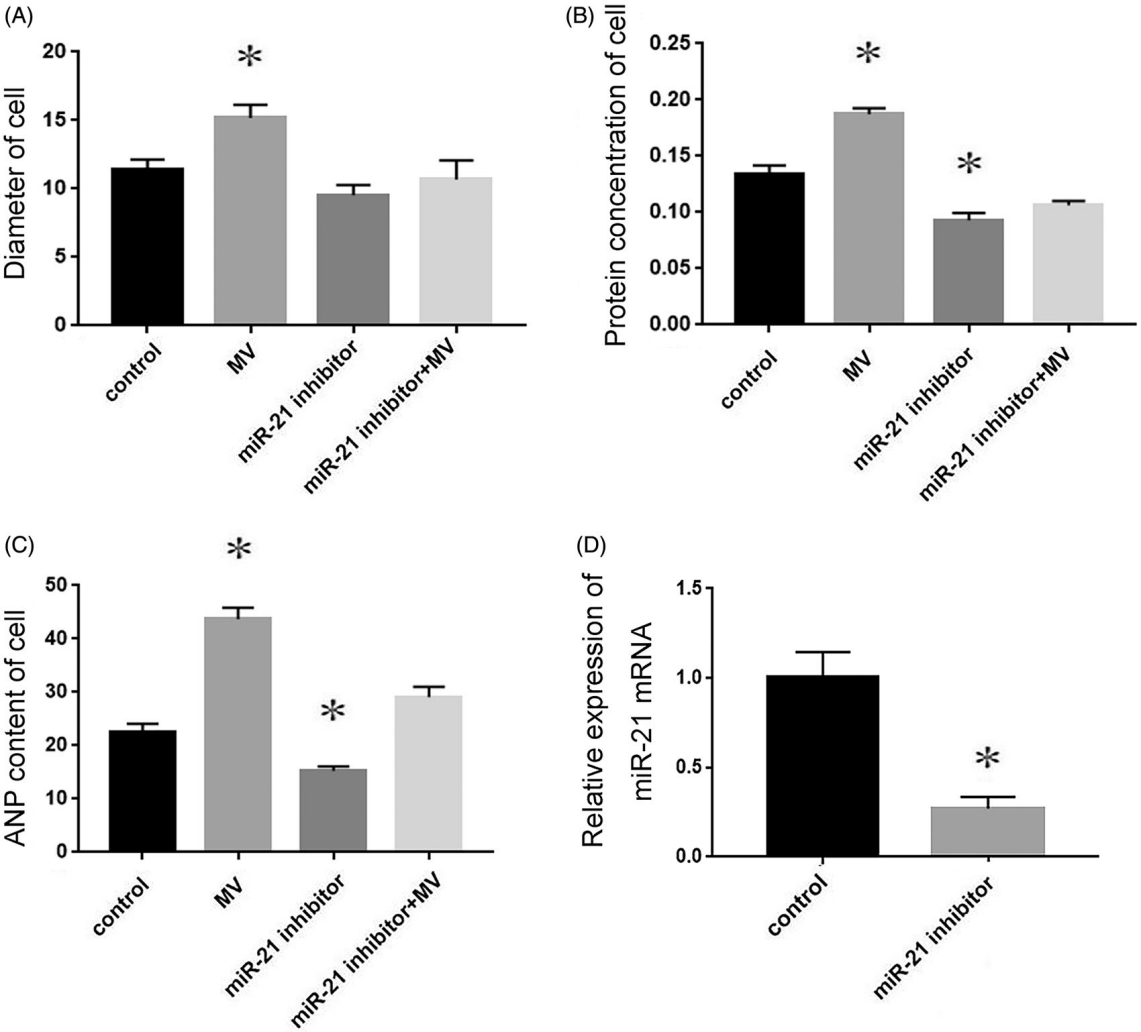
The role of exogenous miR-21 in promoting cardiomyocyte hypertrophy. The diameter of cell (A), protein concentration (B) and ANP content (C) of cardiomyocytes treated with normal medium, conditioned medium containing MVs, miR-21 inhibitor and MVs containing miR-21 inhibitor respectively. The transfection miRNA21 inhibitor efficiency (D) *p < 0.05 versus control.

To determine the role of exogenous miR-21 in promoting cardiomyocyte hypertrophy, miR-21 inhibitor was pre-transfected into recipient cardiomyocytes. Next, MVs were collected from the conditioned medium and incubated with the pre-transfected recipient cells for 48 h. Compared with the cells transfected with NC RNA, cells incubated with MVs isolated from conditioned medium underwent hypertrophy, while cells pre-transfected with miR-21 inhibitor were resistant to MV-induced increase of cell size (Figure 4(A)), protein concentration (Figure 4(B)) and ANP content (Figure 4(C)). These results demonstrated that miR-21 inhibitor almost completely eliminated the cardiomyocyte hypertrophy induced by conditioned MVs. Transfection of miRNA21 inhibitor reduced miR-21 levels by more than 70% compare with cells transtected with a negative control (Figure 4(D)).

### miR-21 targets SORBS2 and ENH in cardiomyocytes

**Figure 5.**
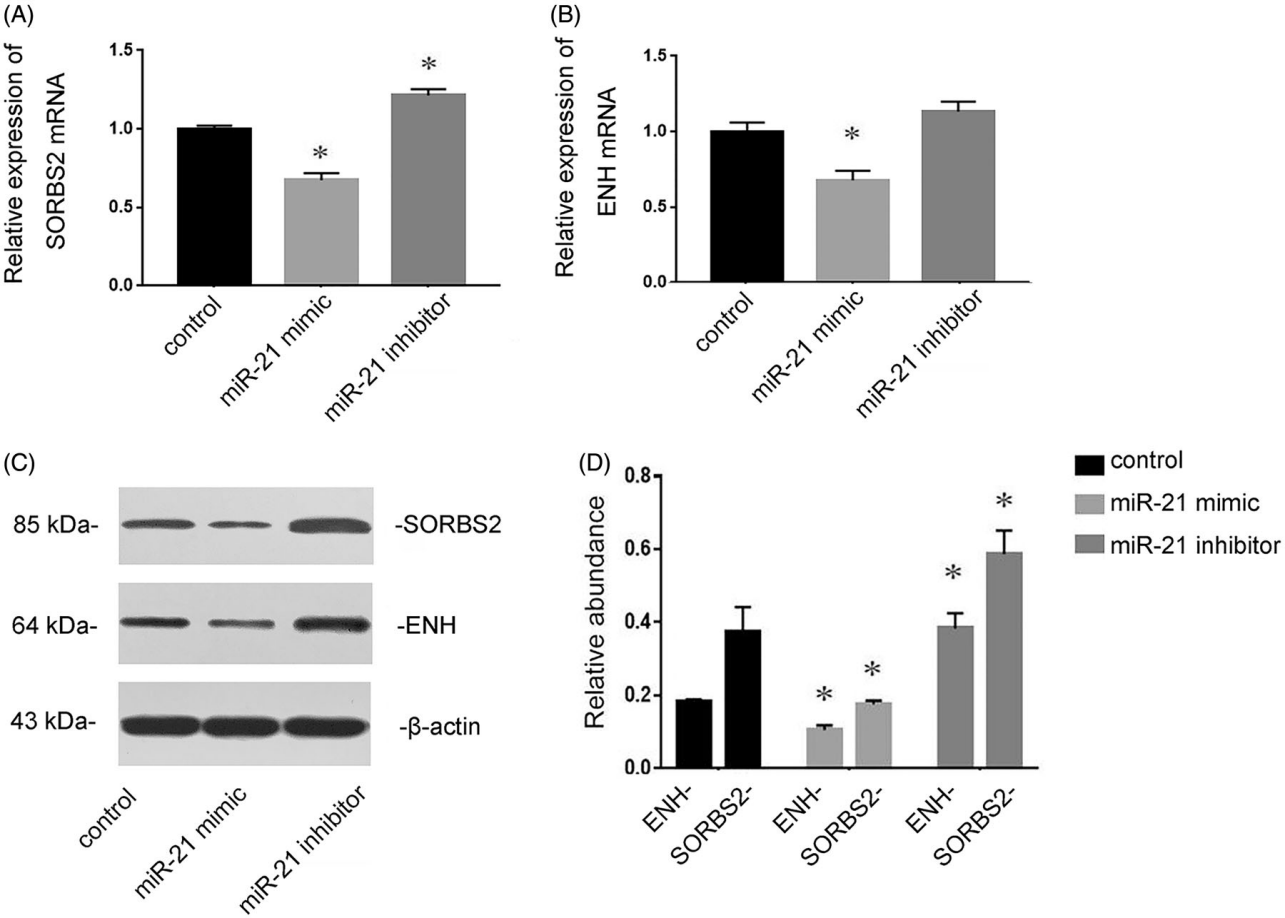
The target genes of miR-21 in cardiomyocytes. Cardiomyocytes were transfected with NC RNA, miR-21 mimic or miR-21 inhibitor for 72 h. RT-qPCR showed that miR-21 mimic inhibited the expressions of SORBS2 and ENH, whereas miR-21 inhibitor induced the expressions of SORBS2and ENH (A and B). Western blotting analysis showed that the expressions of SORBS2 and ENH at the protein level were also inhibited by transfection of miR-21 mimic but induced by miR-21 inhibitor (C and D). *p < 0.05 versus control.

SORBS2 and ENH have been demonstrated to be the targets of miR-21 [9]. The effects of miR-21 on the expressions of SORBS2 and ENH were determined in cultured cardiomyocytes. RT-qPCR revealed that transfection of miR-21 mimic inhibited the expressions of SORBS2 and ENH, whereas pre-transfection of miR-21 inhibitor induced the expressions of SORBS2 and ENH (Figure 5(A,B)). Consistent with the results of RT-qPCR, Western blotting analysis showed that the expressions of SORBS2 and ENH at the protein level were also inhibited by transfection of miR-21 mimic, while they were up-regulated by transfection of miR-21 inhibitor (Figure 5(C,D)).

### miRNA-21 induces cardiomyocyte hypertrophy *via* targeting SORBS2 and ENH

**Figure 6.**
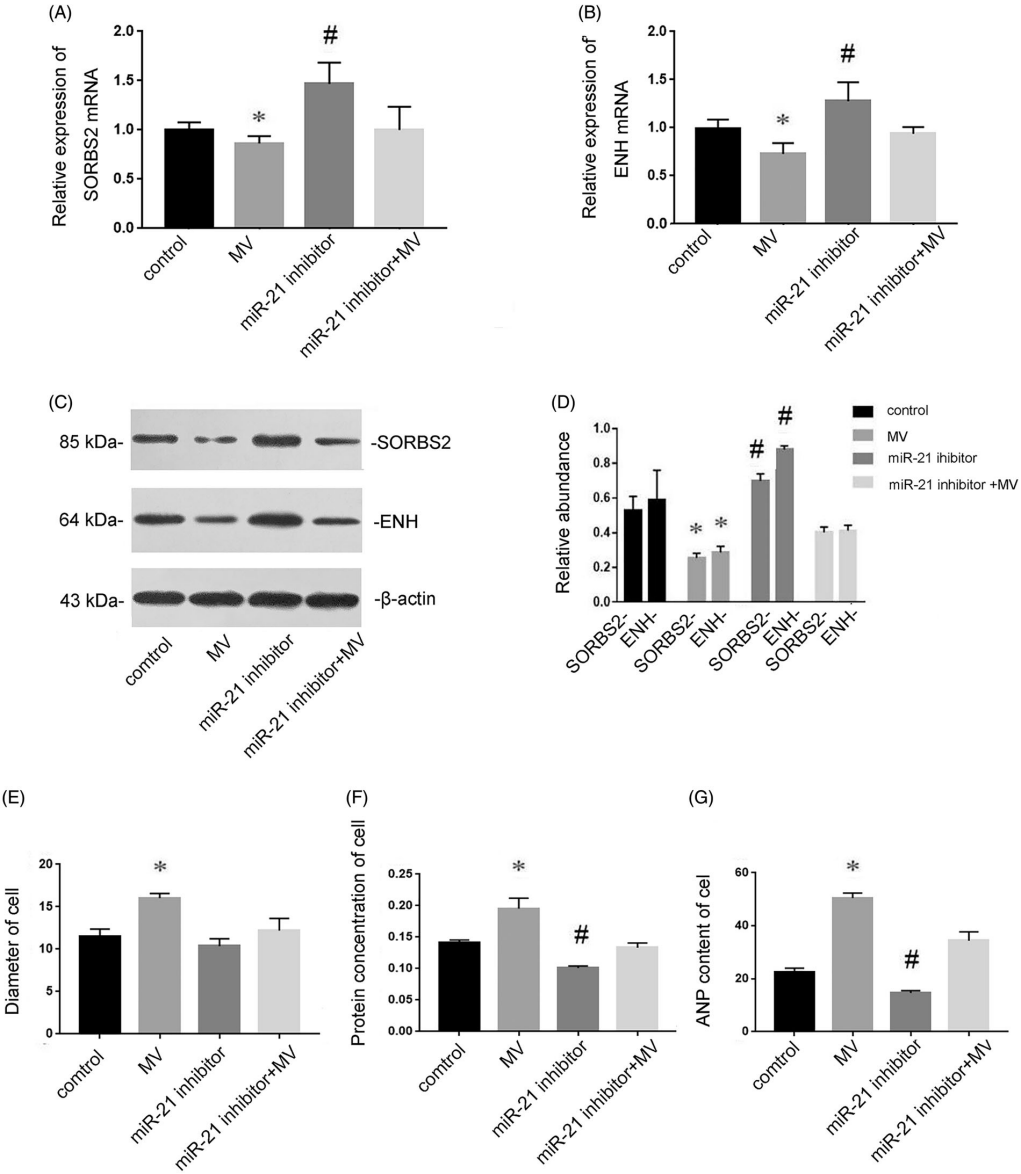
MiR-21 induces cardiomyocyte hypertrophy *via* targeting SORBS2 and ENH. Cardiomyocytes were treated with normal medium, conditioned medium containing MVs, miR-21 inhibitor and MVs containing miR-21 inhibitor, respectively. RT-qPCR showed that the expressions of SORBS2 and ENH at the mRNA level in recipient cardiomyocytes (A and B). Western blotting analysis showed that the expression of SORBS2 and ENH at the protein level (C and D). The diameter of cell (E), protein concentration (F) and ANP content (G) of cardiomyocytes treated with normal medium, conditioned medium containing MVs, miR-21 inhibitor and MVs containing miR-21 inhibitor respectively. Results are presented as means ± SE of three independent experiments. *p < 0.05 versus control. #*p* < 0.05 versus MV.

To further investigate the mechanism underlying the miR-21-induced cardiomyocyte hypertrophy, the expressions of SORBS2 and ENH at the mRNA and protein levels were determined to assess the effects of exogenous miR-21 delivered by MVs in cardiomyocytes. Figure 6(A,B) displayed that the expressions of SORBS2 and ENH at the mRNA level were decreased after incubation with MVs derived from conditioned medium. To exclude the possibility that effects were caused by factors other than miR-21, miR-21 inhibitor was transfected into recipient cells. As we expected, miR-21 inhibitor abolished the depression of SORBS2 and ENH by MVs. Western blotting analysis also showed that the expressions of SORBS2 and ENH at the protein level were decreased after incubation with MVs derived from conditioned medium (Figure 6(C,D)), while transfection of miR-21 inhibitor induced the expressions of SORBS2 and ENH. Furthermore, incubation with MVs derived from conditioned medium resulted in enlarged cell size (Figure 6(E)), increased protein concentration (Figure 6(F)) and elevated ANP content (Figure 6(G)), whereas the hypertrophic effects of MVs were abolished by miR-21 inhibitor. Taken together, these results demonstrated that MV-secreted miR-21 could be delivered into recipient cells, where they induced cardiomyocyte hypertrophy *via* targeting SORBS2 and ENH.

## Discussion

Studies have shown that MV is an important carrier to mediate non-contact cell-to-cell information communication [10,11]. Under normal physiological conditions, cells can produce a small amount of MVs, while such production is significantly increased under some pathophysiological conditions [11]. Interestingly, miR-21 is highly expressed in the heart and kidneys and specifically involved in all types of CRS. Additionally, elevated levels of miR-21 related with a poor outcome. In the present study, we reported that the secreted miR-21 could serve as the molecule mediating kidney and heart communication. It may provide valuable insights into the mechanism underlying the progression of CKD-related CVD.

In the present study, we first induced the injury of renal tubular epithelial cells with TGF-β1 and collected the conditioned medium. MVs were extracted and observed under electron microscope (Figure 1). MV is a microcosm of cell state. In general, cell membrane phospholipids, such as phosphatidylserine (PS) and phosphatidylethanolamine (PE), are located inside of the cell membrane. When the intracellular calcium concentration is increased, PS turns from the inside of the cell membrane to the outside [12]. In this study, donor cells were labeled with fluorescent dye Dil-C18, because Dil-C18 is a type of lipid dye, which can be used to label the cell membrane of donor cells. The lipid membrane structure of MV usually contains the cell membrane from donor cells. When donor cells secrete MVs, the MVs are also labeled with Dil-C18 [8]. Therefore, such labeling can be used to verify whether the MVs produced by donor cells can enter the recipient cells. In our present study, the recipient cardiomyocytes treated with MVs could be labeled with Dil-C18, and the longer of the treatment, the more MVs entered the cells. These results showed that MVs could be transmitted from tubular epithelial cells to cardiomyocytes (Figure 2).

To further verify the effects of these MVs, renal tubular cells were treated with TGF-β1, the culture medium was obtained by ultracentrifugation, and MVs were applied to cardiomyocytes. The results showed that MVs could induce cardiomyocyte hypertrophy, while the culture medium without MVs had no such effect, suggesting that MVs produced by renal tubular cells treated with TGF-β1 were the cause of cardiomyocyte hypertrophy (Figure 3). Although MVs are important vectors for intercellular communication, the substances contained in MVs indeed mediate the interaction between cells. Circulating miRNAs are very stable, which can avoid the degradation of RNase by the protection of MVs, thus attracting a wide range of interests [12]. MiR-21 has been reported to be related to pathological cardiac remodeling and fibrosis [13]. It has been found that MVs from endothelial progenitor cells can antagonize angiotensin II-induced cardial hypertrophy and apoptosis [14], suggesting the participation of MVs in myocardial hypertrophy. Moreover, miR-21 has also been shown to play an important role in renal fibrosis [15]. It has been confirmed that miR-21 regulates peroxisome proliferator-activated receptor alpha, a molecular mechanism of cardiac pathology in CRS type 4 [16]. Suppression of miR-21 protects rats with 5/6 nephrectomy from development of left ventricle hypertrophy and impairment of left ventricle function [16]. It has been also reported that the level of miR-21 in blood circulation is correlated with the severity of renal fibrosis, suggesting that the miR-21 delivered by MVs may enter the blood circulation [17]. Therefore, it is reasonable to speculate that MVs are the inner mechanisms, which form an interaction between heart and kidney diseases. In this study, we observed that myocardial hypertrophy was stimulated by the incubation of MVs. However, such effect could be eliminated by miR-21 inhibitor (Figure 4). Therefore, MVs mediated the delivery of miR-21 into recipient cardiomyocytes, thereafter contributing to the hypertrophy of cardiomyocytes.

Myocardial remodeling is an important pathological mechanism in the progression of heart failure, and cardiomyocyte hypertrophy is one of the characteristics of myocardial remodeling. Arginine binding protein 2 (SORBS2) and ENH are reported to be associated with myocardial hypertrophy [18]. SORBS2 regulates the biological effects of cardiomyocytes, such as the assembly of myofibrils, and depletion of SORBS2 leads to cardiomyocyte hypertrophy [19]. ENH mediates the phosphorylation of L-type calcium channel by protein kinase D (PKD) 1, and the over-activation of PKD results in myocardial hypertrophy. ENH–deficient mice manifest as myocardial contractile dysfunction and dilated cardiomyopathy [20]. According to the prediction of Targetscan and results of previous studies, it is found that SORBS2 and ENH are the target genes of miR-21. Therefore, we examined the role of miR-21 contained in MVs on SORBS2 and ENH in the progression of myocardial hypertrophy. Figure 5 shows that the expressions of SORBS2 and ENH were inhibited by transfection of miR-21 mimic, while they were induced by miR-21 inhibitor, indicating that SORBS2 and ENH were the target genes of miR-21 in cardiomyocytes. Our further investigation found that miR-21 presented in MVs could induce cardiomyocyte hypertrophy *via* targeting SORBS2 and ENH (Figure 6).

Recently, more and more studies suggested that miR-21 is an independent risk factor for CRS and has been regarded as a key therapeutic target [21,22]. MiR-21 is upregulated in cardiac or renal diseases and can also be transported by microvesicles to other cell types [2]. Suppression of miR-21 could significantly improve the cardiac and renal function and may represent an attractive therapeutic option. In the current study, we identified a novel MV-mediated communication mechanism between renal tubular epithelial cells and cardiomyocytes. Taken together, our results indicated that miR-21 was exported from renal tubular epithelial cells *via* MVs, contributing to the development of cardiomyocyte hypertrophy, these findings might provide new mechanism and therapeutic targets in CKD-related CVD.

There are a few limitations in this study. First, there were only *in vitro* experiments, data of animal experiments and human specimens are required in further work. In addition, it would be more convincing by using more experimental methods.

## Data Availability

All data generated or analyzed during this study are included in this published article.

## Abbreviations

CVD: cardiovascular disease
miR-21: miroRNA-21
MV TGF-β1: microvesicle transforming growth factor
CKD: chronic kidney disease
miRNA: microRNA
PS: phosphatidylserine
PE: phosphatidylethanolamine
ENH: Enigma homolog
PKD: protein kinase D
